# Inequalities, harm reduction and non-combustible nicotine products: a meta-ethnography of qualitative evidence

**DOI:** 10.1186/s12889-020-09083-9

**Published:** 2020-06-15

**Authors:** Mark Lucherini, Sarah Hill, Katherine Smith

**Affiliations:** 1 School of Geography, Geology and the Environment, Keele University, Keele, UK; 2 Global Health Policy Unit, School of Social & Political Science, University of Edinburgh, Edinburgh, UK; 3 School of Social Work & Social Policy, University of Strathclyde, Glasgow, UK

**Keywords:** E-cigarettes, Smokeless tobacco, Nicotine replacement therapy, Inequalities, Qualitative research; socioeconomic status

## Abstract

**Background:**

We sought to review qualitative evidence on how smokers in different socioeconomic groups engage with non-combustible nicotine products (NCNP), including electronic cigarettes and nicotine replacement therapies, in order to provide insight into how these products might impact on smoking inequalities.

**Methods:**

We searched ten electronic databases in February 2017 using terms relating to NCNP and socioeconomic status. We included qualitative studies that were published since 1980 and were available in English. We used guidelines adapted from the Critical Appraisal Skills Programme for appraising qualitative research.

**Results:**

The review only identified studies exploring the attitudes of socioeconomically disadvantaged smokers towards NCNP for harm reduction or cessation purposes (i.e. we did not identify any relevant studies of more advantaged socioeconomic groups). Using a lines-of-argument meta-ethnographic approach, we identified a predominantly pessimistic attitude to NCNP for harm reduction or cessation of smoking due to: wider circumstances of socioeconomic disadvantage; lack of a perceived advantage of alternative products over smoking; and a perceived lack of information about relative harms of NCNP compared to smoking. Optimistic findings, although fewer, suggested the potential of NCNP being taken up among smokers experiencing socioeconomic disadvantage.

**Conclusions:**

Overall, our review highlights the importance of considering the social, cultural and economic circumstances that influence experiences of smoking and of alternative product use.

## Background

The recent emergence of e-cigarettes in the nicotine market has rejuvenated debates on tobacco harm reduction and cessation. Some hail the devices as game changers in the struggle to reduce smoking prevalence [1], while others debate their ability to positively impact on existing smoking inequalities [2–5]. Meanwhile, quantitative data continues to demonstrate a concentration of smoking within socioeconomically disadvantaged communities across Europe [6]. A recent systematic review of quantitative evidence, conducted by the authors of this review, found limited evidence that non-combustible nicotine products (NCNP) had reduced or are likely to reduce socioeconomic inequalities in smoking [7]. The quantitative review included all types of NCNP, including e-cigarettes, in order to explore differential potential for reducing inequalities of the wide range of products that now exists. However, these quantitative studies tell us little about how users themselves perceive and experience NCNPs. Qualitative insights are likely to be useful in not only understanding why quantitative trends appear the way they do but whether and how this might be open to change. Therefore, using a meta-ethnographic approach, this study reviewed qualitative data on attitudes towards all NCNP to help understand how social context informs the significance of practices of use of NCNP.

Our quantitative review considered multiple indicators of relative socioeconomic status, including income, education, occupation and class. In this qualitative review, as we explain below, all of the relevant studies we identified focused on groups that were socioeconomically disadvantaged in some way. We use the term socioeconomic disadvantage to refer to multiple intersecting indicators such as income, education, occupation and class. Included studies sometimes used different terms and indicators of socioeconomic disadvantage so we explain these as they occur. The review was not intended to identify other studies using indicators of social or cultural advantage or disadvantage such as race, ethnicity, gender or age.

In the context of tobacco control, ‘harm reduction’ refers to strategies that support reduced use of tobacco – ranging from stopping smoking to cutting down and temporary abstinence – with NCNP often being presented as aids to achieve such harm reduction [8]. There is evidence that those who use one such set of products – nicotine replacement therapies (NRT) – to reduce smoking are more likely to eventually quit than those who do not [9], lending support to harm reduction as a first step in ultimately quitting altogether. Historically, in the UK, only NRT – including gum, patches, lozenges, and inhalers – have been officially endorsed as aids to smoking reduction or quitting [8]; but more recently, a number of UK organisations have promoted e-cigarettes as harm reduction products [10–13]. While e-cigarettes are a relatively new technology compared to NRT products, early evidence from the UK points to their use being associated with an increased number of quit attempts [14], leading some experts to postulate that increasing e-cigarette use in the population will eventually drive down smoking prevalence [15]. Other scholars have been careful to not promote e-cigarettes in the context of uncertainties about potential impact on youth uptake [16], the related possibility that e-cigarettes might act as a ‘gateway’ to smoking [17], and the rejuvenation of tobacco industry policy influence in the context of increasing ownership of e-cigarettes companies by the tobacco industry [18].

More optimistically, some commentators working in public health have suggested that e-cigarettes have potential for addressing socioeconomic inequalities in smoking, noting their relative low-cost (compared to regular purchases of cigarettes) and wide availability [4, 10]. Yet, there is no clear evidence to date to indicate that e-cigarettes will contribute to reducing socioeconomic inequalities in conventional tobacco use and some scholars have suggested they may actually exacerbate inequalities in smoking due to the higher cost of more sophisticated and advanced product types [2, 5]. Even ‘affordable’ e-cigarette starter kits (~£10) can be too expensive for those experiencing socioeconomic disadvantage [19]. This has been supported by recent research that suggests the most effective e-cigarettes for cessation are the most expensive [20]. There are also fears that the tobacco industry may deliberately exploit this by promoting basic low-efficacy models [21]. Extensive evidence also suggests that socioeconomically disadvantaged smokers find it more difficult to quit smoking, even where motivation and support levels are comparable with socioeconomically advantaged smokers [22]. For example, previous research has shown that cessation support with conventional NRT has lower efficacy among socioeconomically disadvantaged smokers than those from more advantaged groups [23, 24]. While there has been much research on the efficacy of NRT for smoking cessation, relatively few studies have examined its impact among disadvantaged populations or explored the experiences and perceptions of these communities [25]. Multiple forms of nicotine use have also created complex patterns of transitions and poly-use among different groups that do not necessarily lead to quitting smoking behaviours, for example dual use of cigarettes and e-cigarettes [26] or transitioning from cigarettes to roll-you-own to reduce costs [27].

Qualitative research is crucial for understanding attitudes towards, and experiences of, tobacco use, harm reduction and cessation. Socioeconomically disadvantaged circumstances often go hand-in-hand with increased smoking prevalence and more ‘normalised’ attitudes towards smoking [28, 29]. Circumstances of stress caused by socioeconomic disadvantage have been cited as reasons for smokers to continue smoking and to avoid, delay, or relapse from cessation attempts [30, 31]. Stead et al. [32] and Thompson et al. [33], have explored how place of residence can isolate disadvantaged communities from wider social norms, in which smoking is denormalised, creating smoking ‘islands’ within countries. Smoking in these communities often becomes connected to social and cultural identity and practices and, as Robinson and Holdsworth [34] find, smoking and cigarettes become shared practices and goods laden with emotional significance. A recent meta-ethnography exploring public understandings and experiences of health inequalities found that smoking was, like other unhealthy behaviours, consistently described by participants experiencing disadvantage as a mechanism for ‘coping with’ or momentarily forgetting difficult and stressful life circumstances [35].

### Aims of the meta-ethnography

Smoking prevalence is relatively high and cessation relatively low in socioeconomically disadvantaged groups within high-income countries [36]. Moreover, qualitative research has regularly demonstrated distinct differences in attitudes to smoking by socioeconomic status (SES). In this context, this review set out to explore how perceptions and experiences of NCNPs varied for different socioeconomic groups. However, since all of the relevant studies we identified focused on groups experiencing socioeconomic disadvantage, the findings are more accurately described as capturing how the perceptions and experiences of socioeconomically disadvantaged groups in using NCNP map onto harm reduction strategies. We used a meta-ethnographic ‘lines-of-argument’ approach to synthesise qualitative studies identified from a larger systematic review of NCNP and use by SES in high-income countries with advanced tobacco control policies. Hence, all included studies in this meta-ethnography focused on examining experiences within contexts of declining tobacco use and policy efforts to further reduce smoking.

## Methods

This review is part of a larger project and the results of a literature review on quantitative data are reported in a separate paper [7]. A full protocol of this project, including the quantitative and qualitative reviews, has been registered with PROSPERO (ID: CRD42017080672) [37]. The review is reported in line with Preferred Reporting Items for Systematic Reviews and Meta-Analyses (PRISMA) Equity (PRISMA-E) guidelines [38] (Supplementary File).

### Search strategy

A search string that used terms for NCNP, SES and combustible tobacco smoking (Supplementary File) was used to search the following 10 electronic databases on 9th February 2017: BIOSIS Citation Index, web of Science Core Collection, Cochrane Library, ProQuest Social Sciences premium collection, CINAHL Plus, EMBASE, Medline (+ Medline Epub ahead of print), PsycInfo, Global Health. An initial 24,711 studies were identified across all databases.

### Study selection

**Fig. 1:**
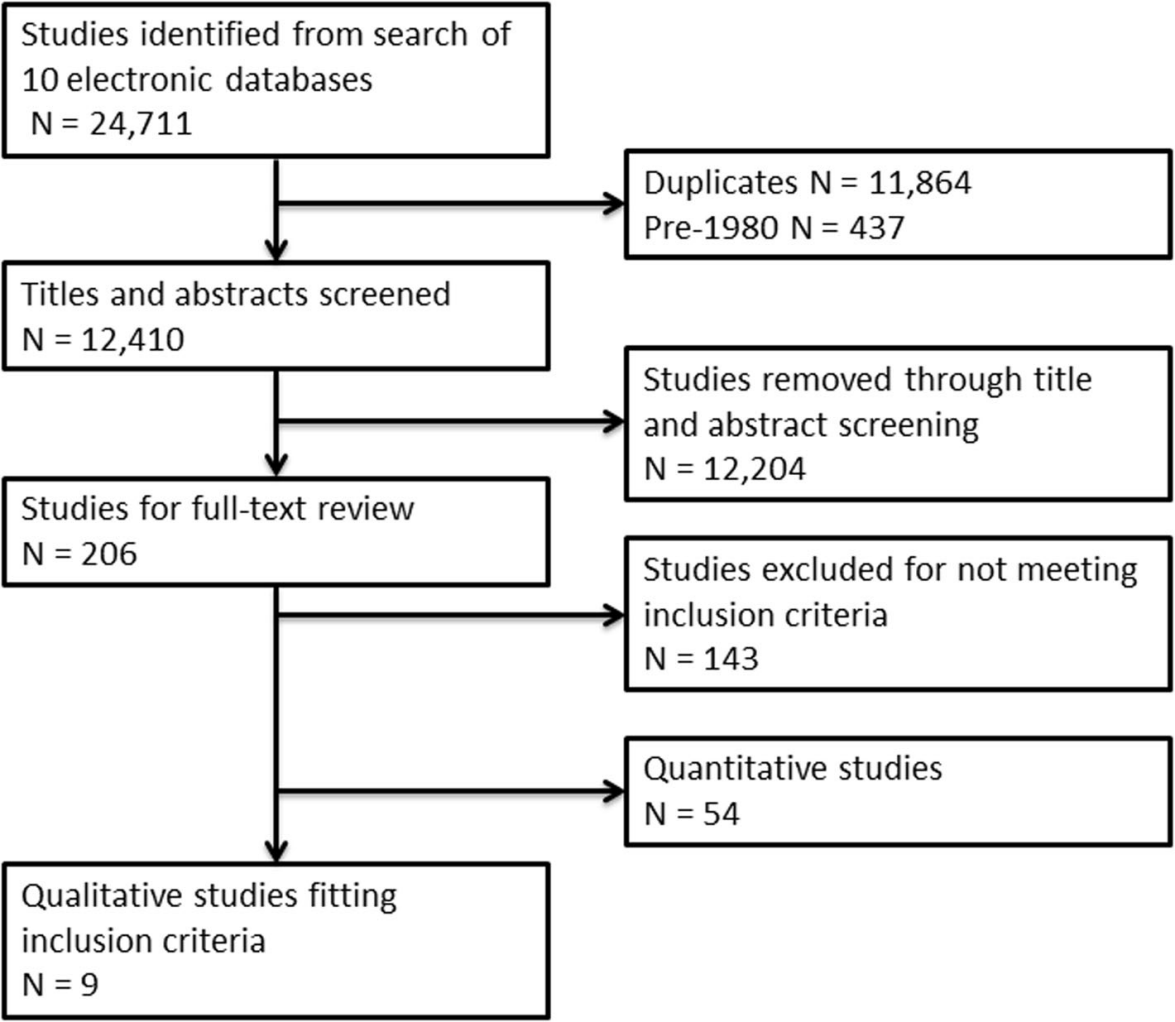
PRISMA flow diagram

Once duplicates had been removed, title and abstract screening identified studies from high-income countries at Stage 4 of the cigarette epidemic [39] which had an NCNP as a the main focus or intervention. Only studies from 1980 onwards were included as this is considered the point at which high-income countries began seriously exploring harm reduction as part of tobacco control [40–42]. Socioeconomic status was defined as any measure (qualitative or quantitative) relating directly to financial circumstance, including income, education, occupation and class. Studies were not limited by smoking status of participants but all identified qualitative studies but had to contain data on the use of at least one form of NCNP. Title and abstract screening was completed by ML and 206 studies were identified. ML carried out the full-text review of these studies and a sample of 25% of studies was independently assessed by KS and SH. All studies excluded from the 206 were reviewed by at least two authors. Ultimately, nine qualitative papers were identified for inclusion in the review (Fig. 1). Three of these papers reported findings on NRT from the same, single study. As the three studies contained significant overlap and two had very limited evidence of use or perceptions of NRT, we elected to analyse them as a single study. We conducted further hand searching of reference lists of included studies but found no further articles to include.

### Data extraction and quality appraisal

A data extraction form was developed and piloted to extract the following information from studies: study design, location, participant characteristics, sample size, study period, type of NCNP and measure(s) of SES. Short textual entries were made to indicate the study’s main findings for the review and what these results might suggest about equity outcomes. Data were extracted by ML and checked by the other authors. Criteria for critical appraisal were created based on the Critical Appraisal Skills Programme (CASP) guidelines for qualitative research [43]. Criteria covered the primary focus of the paper, appropriateness of study design, sample recruitment, methodology, analysis and generalizability. All papers were considered of adequate quality to be included in the review (see Supplementary File).

### Data analysis and synthesis

Noblit and Hare [44] categorised three different types of meta-ethnography analysis: lines-of-argument, reciprocal and refutational. We selected a lines-of-argument approach (in which studies are directly translated into one another), which enabled us to consider what we can say about the ‘whole’ based on selective studies of the ‘parts’. We felt this approach was suitable given the similar number of studies identified for each NCNP type (although there were six studies of NRT, three of these draw from the same data-set and so are counted as a single ‘study’) [45–47]. In contrast, we felt that reciprocal analysis assumed too much similarity between different forms of NCNP, while a refutational analysis (which focuses on contradiction between studies) assumed too much difference. Nonetheless, there were some important contrasts within the studies and we consider these in the discussion section. We discerned our lines-of-argument from recurring themes across the studies that indicated barriers or facilitators to NCNP uptake and perceptions of NCNP in relation to harm reduction.

The synthesis was completed in five stages (Table 1). In the first stage all papers were read by all three authors. In the second stage we categorised the included studies by NCNP type (e.g. NRT, e-cigarettes), following a similar approach of separating distinct product categories for analysis in a meta-ethnography of taking medicine for asthma [48]. The different study categories were coded independently of each other, given the difference in the length of time these products have been available and their different modes of use. This enabled us to examine the differences in findings between NCNPs, which we felt was important given they involve different products, forms of use and availability. Once categorised into sub-groups, the findings/results sections of each study were coded line-by-line. Reflecting our lines-of-argument we did not approach each study as a whole but sought to disassemble individual studies into codes and then reassemble all the codes together to achieve an outcome which provides a new, more holistic perspective on the topic [48]. First order codes of participants’ accounts and second order codes of authors’ interpretation were coded separately in NVivo 10. We took first order data to refer to participants’ own words (quotations) or direct descriptive summaries of participants’ own words by authors. We took second order data to refer to interpretations and summaries by authors of participant perceptions and attitudes. In the third stage, meta-themes were created by ‘translating’ codes for both the first order and second order across the grouped studies. In most cases the second and first order translations reflected each other, providing assurance that we had interpreted the data in a similar way as the authors (Supplementary File).

**Table 1:** Synthesis stages

**Stage**	**Process**
First	In-depth repeated reading of studies.
Second	Creation of study sub-sets by NCNP type and line-by-line coding of first and second order themes.
Third	Translation of first and second order themes within grouped studies to create ‘meta-themes’.
Fourth	Creation of lines-of-argument informed by research questions within grouped studies.
Fifth	Translation of lines-of-argument across NCNP types.

In the fourth stage, further translation of codes was derived from the meta-themes through an application of our study aims to create lines-of-argument – which summarised reasons why people would use NCNP, would not use NCNP and how they saw NCNP as part of harm reduction strategies. In the fifth and final stage, we translated the findings across NCNP type to create a final lines-of-argument synthesis.

## Results

### Study summary

There were nine included studies, three of which focused on e-cigarettes (Table 2) and six on NRT (Table 3). We did not identify any qualitative studies of smokeless tobacco use that had a focus on distinct SES categories. Most participants across the studies were either smokers or ex-smokers. All included studies focussed specifically on groups that were socioeconomically disadvantaged in some way. We found no eligible studies that focussed on clearly identifiable groups of other SES, suggesting there is gap in existing research in terms of qualitative research that looks across SES groups or which focuses on understanding experiences and perceptions of NCNPs in more advantaged groups. Most studies recruited from areas of the UK deemed to be either socioeconomically disadvantaged or ‘working class’ [29, 49–51, 53], a term commonly used in the UK to describe lower SES groups [54]. One study [52] used the UK Townsend Deprivation Index, while three others [45–47] (from the same dataset) recruited participants from welfare service users in Australia. Many studies identified in the original search were not included on the basis that they did not contain references to NCNP or were focussed on some other aspect of disadvantage that was not related to SES.

**Table 2:** Included e-cigarettes studies

Study	Location	Participants	Methods	SES	Study period	Study aims
Rooke et al. 2016 [49]	Central Scotland	*N* = 64, smokers and recent ex-smokers, mean age 36, 33 female	Interviews and focus groups	Recruitment from socially disadvantaged areas	2013–2014	To explore the understandings and experiences of e-cigarettes among disadvantaged smokers and recent ex-smokers
Rowa-Dewar et al. 2017 [50]	Five communities in Edinburgh, UK	*N* = 25, smokers and some ex-smokers, parents of young children, 22-47yo, 22 female	Interviews	Recruitment from socially disadvantaged areas	2013–2014	To explore the uses and perceptions of e-cigarettes by disadvantaged parents, especially in relation to temporary smoking abstinence in the home.
Thirlway 2016 [29]	North West Durham, North-East England, UK	*N* = 41, smokers and ex-smokers, 18-75yo, mean age 42, 28 men	Ethnographic observation, including field notes and interviews	Recruitment from predominantly working class sites	2012–2015	To explore the potential of e-cigarettes to address health inequalities.

**Table 3:** Included NRT studies

Study	Location	Participants	Methods	SES	Study period	Study aims
Atkinson et al. 2013 [51]	Nottingham, UK	36, smokers, parents of young children, 16yo and over, 28 female	Interviews	Recruitment from socially disadvantaged areas	2009	To explore the uses and perception of NRT by disadvantaged parents, especially in relation to temporary smoking abstinence in the home.
Bonevski et al. 2011 [45] Bryant et al. 2010 [46] Bryant et al. 2011 [47]	New South Wales, Australia	32, smokers, 16yo+, 22 female	6 focus groups of 3–8	Users of community welfare services	2008–2009	To explore the barriers and opportunities for smoking cessation for disadvantaged smokers.
Roddy et al. 2006 [52]	Nottingham,UK	39, smokers, 27-77yo, 33 male	9 Focus groups of 2–7	Local indicator of SES (Townsend score)	Unclear	To identify barriers or motivators among disadvantaged smokers to accessing smoking cessation services.
Wiltshire et al. 2003 [53]	Two sites in Edinburgh, UK	100 smokers, 25-40yo, 50 female	Interviews	Recruitment from socially disadvantaged areas	1999–2000	To investigate the perceptions and experiences of quitting among smokers from disadvantaged areas

Although the studies contained relevant data for either e-cigarettes or NRT and there was only some overlap in discussion between those two types of NCNP in any study, the ultimate lines-of-argument analysis for each NCNP were relatively similar with many themes occurring in both strands of analysis and being straightforwardly translated into each other. For this reason we have largely considered NRT and e-cigarettes together (where this was not the case, we are explicit in the results). The studies rarely differentiated between the different types and models available of e-cigarettes (e.g. cigalike, tanks/mods) or NRT (e.g. gum, patches, inhaler). Hence, by necessity, we also had to treat each NCNP type largely collectively. However, it should be noted that e-cigarettes and NRT are both broad categories and the types of product available for both are becoming increasingly diverse [20, 55].

We categorised our final lines-of-argument into three overall views on the prospects of NCNP for harm reduction: ‘pessimistic’, ‘optimistic’ and ‘uncertain’ (Table 4). Full details of the coding process can be found in the Supplementary File. Pessimistic results, which reflected a general lack of enthusiasm or perceived ability to use NCNP to replace smoking, were the most common results and so are discussed first. Optimistic findings, which relayed enthusiasm for using NCNP to replace or reduce smoking, were the next most common result so are discussed next. Finally, we discuss the least common category; views and experiences that seemed ‘uncertain’ about NCNP, especially in terms of the potential for harm reduction or cessation. We note, at the outset, that there were significantly more optimistic findings relating to e-cigarettes than to NRT, which appeared to reflect different modes of use and understandings of relative harm.

**Table 4:** Final lines-of-argument synthesis

**Pessimistic (more common findings)**	**Uncertain (more common findings)**	**Optimistic (less common findings)**
Social, cultural and economic circumstances of socioeconomically disadvantaged smokers not conducive to NCNP uptake	NCNP positioned as useful for smoking reduction but not necessarily smoking cessation NCNP alone perceived to have limited potential for smoking harm reduction	Social, cultural and economic circumstances of socioeconomically disadvantaged smokers can be conducive to NCNP uptake
NCNP do not carry enough ‘relative advantage’ over smoking		NCNP have some ‘relative advantage’ over cigarettes
Lack of clear information about relative harm of NCNP		Accepted knowledge about relative harm and NCNP

### Pessimistic views

This section discusses the pessimistic views towards NCNP that were identified across the studies, focusing on: (i) how social, cultural and economic circumstances often seemed to deter NCNP use; (ii) explaining why NCNP were considered as having little relative advantage over smoking; and (iii) perceptions of a lack of information about NCNP.

*(i) Social, cultural and economic circumstances of socioeconomically disadvantaged smokers are not conducive to NCNP uptake.* Social and cultural circumstances in which smoking was perceived as a normal part of everyday life was a feature of most studies. Stress was a common experience mentioned by participants across the studies and some appeared to feel resigned to returning to smoking after periods of exclusive e-cigarette use. For example, a participant from Rooke et al. [49: e63] talked about switching back to combustible tobacco, attributing this directly to experiencing stress:

Because [ … ] there’s a wee bit too much stress in my life at the moment that I had to go back on the cigarettes. (Female, 47, smoker)

The reliability of combustible tobacco compared to NCNP, and its retention as a last resort, was similarly evident in a participant from Thirlway’s [29: 109] study, who was;

A serial quitter and had tried everything to give up; she bought an e-cigarette … and this went well until “it rolled off the table and broke” and she reverted to the pouch of illicit rolling tobacco tucked into the front pocket of her tabard

Stress therefore made it difficult for NCNP to take hold for many of the participants in the studies.

Gender appeared to intersect with socioeconomic disadvantage to shape experiences in several studies. For example, female participants in Thirlway’s study [29] frequently described prioritising family caretaking over their own wellbeing, leaving little time for self-care, such as smoking reduction. Additionally, Thirlway [29] noted that e-cigarette use in a working class area in north-east England appeared to be dominated by young men who appeared to be attracted to the novelty and gadgetry of the devices, with some women seeming reluctant to visit vape shops where young men congregated.

Thirlway [29] also identified a ‘working-class hedonism’ throughout her study, e.g. “them at Greendale [middle class area] haven’t enjoyed themselves the way us lot have – I’ve no regrets” (p. 110, male, 47, smoker). Thirlway observed that other young men in her study felt vaping did not fit with a ‘hedonistic’ masculinity that valued carefree consumption. Hence, there were variations in local responses to, and views of, e-cigarettes, both between men and women and among men.

Experiences of unemployment also influenced attitudes towards NCNP. Atkinson et al. [51] and Wiltshire et al. [53] noted that participants rarely encountered situations of enforced temporary abstinence at home or work due to unemployment. For example a male (smoker) participant in Wiltshire et al. said of him and his partner: “[We’re] not comfortable living here... I’m unemployed... Stress levels have been very high [and] we have noticed we smoke a lot more” (p. 299). Atkinson et al. and Wiltshire et al. both suggest that encountering such restrictions is more common for more affluent individuals in higher grades of employment, and for whom smoking reduction and cessation then becomes expected and normalised.

The perceived high price of NCNP, along with a concern about not getting value for money if they turned out not to help smoking cessation or reduction, recurred throughout most of the studies. Financial concerns were particularly highlighted by participants in Thirlway’s [29] study:

Although £10 would buy a starter tank and e-liquid, smokers like Martin could get a week's worth of illicit rolling tobacco for the same money and could not risk such a large outlay on something that might not ‘work’ for him (p. 108).

Furthermore, Thirlway noted that people were likely to revert to cigarettes when their e-cigarette broke, rather than seek a replacement (as illustrated in earlier extract). Similarly, Wiltshire et al. [53] and Roddy et al. [52] found that cigarettes were easily obtainable through informal networks when money was tight, suggesting that the financial disincentive to smoke was not as great as might be expected in a context of high tobacco taxation.

*(ii) NCNP do not carry enough ‘relative advantage’ over smoking or other harm reduction products.* When discussing NRT, many of the participants explained that it had an unpleasant taste – reflecting findings by Dawkins et al. [19] – or said that they felt it did not work as intended. Additionally, some participants explained that they enjoyed smoking and were unconcerned about continued smoking.

I know it’s bad for me and everything like that, but I do enjoy it. (Female, smoker) (Wiltshire et al: 297 [53])

I just like fags. I just like the taste of fag. (Male, 20, smoker) (Rooke et al: 063 [49])

While NRT represented a complete break from smoking actions in ways that sometimes seemed incompatible with participants’ stress relief rituals, e-cigarettes were sometimes experienced as unsettling precisely because of their similarity to smoking. For example, some participants noted that switching to an e-cigarette did not feel like quitting and that the similarities could potentially continue an addiction and/or habit that was perceived negatively:

I don’t feel like I’ve stopped smoking, I just feel like I smoke them instead. (Female, 47, ex-smoker) (Rooke et al: e63 [49])

It’s not getting rid of the habit. [...] I’m still trying to persuade my husband to go on [nicotine replacement] patches, because I’m like, honestly, you’ve got to stop with that part of it [simulating smoking action]. (Female, 40, smoker) (Rowa-Dewar et al: 18 [50])

Likewise, Thirlway [29] found that some people regarded addiction as the primary ‘deviance’ so expressed some unease about e-cigarettes, given the continuation of nicotine use.

*(iii) Lack of clear information about relative harm of NCNP.* Relatedly, many of the studies reported uncertainty about the relative harms of NCNP, often centring on the continuation of nicotine consumption and the potential of becoming addicted to something new.

I kind of understand it [NRT] … but then on the other side of it I think because it’s nicotine replacement so how is it gunna help you stop if it’s still giving you the nicotine. (Female, 25-34, smoker) (Atkinson et al 2013: 4 [51])

Studies on e-cigarettes also found some participants to be unsure about the health risks of e-cigarettes.:

I don’t trust the electronic cigarettes, I just...I don’t think there’s been enough research on them. (Male, 39, smoker) (Rowa-Dewar et al: 17 [50])

Some of the participants in Rooke et al. [49] were particularly distrustful of e-cigarettes that were not sold through official retailers such as *Boots,* citing unknown and possibly dangerous ingredients. Atkinson et al. [51] believed that their data suggested the negative effects of environmental tobacco smoke had come to be underestimated by participants due to a lack of knowledge, and contributed to lack of uptake of less harmful NRT in the homes of those smokers with children.

### Optimistic views

Despite a general lack of enthusiasm for all forms of NCNP across the nine studies, there were also some more positive attitudes to e-cigarettes within all studies. These attitudes were informed by: (i) accounts of social, cultural and economic circumstances; (ii) perceptions of the relative advantages of NCNP; and (iii) accepted knowledge about the reduced harms of NCNP.

*(i) Social, cultural and economic circumstances of socioeconomically disadvantaged smokers can be conducive to NCNP uptake*. In contrast to e-cigarettes not fitting with a ‘hedonistic’ identity, their novelty technology also appeared to be an attractive point for some young men who used the devices to develop a ‘vaper’ identity through expertise and owning the latest equipment:

When last I saw Adam (30, smoker), he was very proud of his latest, fourth-generation e-cigarette with wireless connectivity, and he told me that several of his friends had followed his example. (Thirlway: 108 [29])

By examining the everyday tactics of buying and using e-cigarettes in a working class community, Thirlway discovered that some smokers used the ‘informal e-cigarette economy’ to avoid higher prices and so resist the more middle-class lifestyle and hobbyist approaches to e-cigarette use. These working class vapers were able to cast their use as functional rather than recreational and so “demonstrate moral worth in relation to the moral problems of addiction and expenditure on the self” (p. 111).

This evidence of a thriving informal economy in Thirlway’s paper indicates the importance of community-led distribution and exchange mechanisms for e-cigarettes and associated items such as e-liquids, which has emerged due to the social aspects of vaping [56]. Aspects of Rooke et al’s study [49] refute this, indicating that smokers prefer ‘trustworthy’ high street retailers and avoid informal retail sources. These differences may be explained in part by the recruitment methods in the two studies. Many of the participants in Rooke et al. were recruited through smoking cessation groups and so may have been predisposed to e-cigarette products that followed licensed NRT in being ‘official’ and endorsed by reputable retailers. In contrast, Thirlway’s participants were approached through general community settings and so not necessarily interested in cessation.

Gender dynamics were further evident in Thirlway’s [29] study as men with serious health problems were able to enrol e-cigarette use in “local constructions of masculinity” through being a “badge of moral intent” (p. 110) to take responsibility for improving health outcomes. Despite this intent, Thirlway observed these men continuing to smoke, or at least being in possession of smoking paraphernalia, and was unsure whether their vaping went beyond a marker of moral identity to signify significant behaviour change.

In contrast to findings suggesting NCNP were too expensive [29, 52, 53], Rowa-Dewar et al. [50] found one instance of e-cigarette use being described as saving money for a couple compared to smoking.

*(ii) NCNP have some ‘relative advantage’ over cigarettes.* Also in contrast to pessimistic findings in the papers about the embodied similarities of smoking and vaping impeding use, Rooke et al. [49] and Rowa-Dewar et al. [50], both found that the embodied similarities between vaping and smoking could also be viewed positively. For one of Rooke et al’s [49] participants:

They’re more satisfying. Much more satisfying. I think because, see when you take a puff, it actually feels like, you used to get that kind of hit off a cigarette when you took a puff off the cigarette, you get that sensation from the e-cig. (Female, 42, ex-smoker) (p. e62)

*(iii) Accepted knowledge about relative harm and NCNP.* Further contrasting aspects within included studies were that Rooke et al. [49] and Rowa-Dewar et al. [50] found some participants to be well informed about the relative harm of vaping compared to smoking, so suggested that e-cigarettes carried a clear relative advantage and were generally healthier than smoking:

You’re still smoking nicotine, but you’re not smoking tar and you’re not making your lungs … you’re not making your lungs get covered in tar. (Male, 20, smoker) (Rooke et al.: e63 [49])

This suggests that the similarity of the experience of vaping to e-cigarettes is viewed as both an attraction and a draw-back, depending on the outlook and preferences of the smoker.

### Uncertain views

Uncertain views of NCNP were characterised by: (i) NCNP being useful for cutting down on smoking but not necessarily stopping; and (ii) NCNP have limited potential for cessation if other drivers for cessation were not present.

*(i) NCNP positioned as useful for smoking reduction but not necessarily smoking cessation.* Both the e-cigarette and NRT studies reported that users felt the products could be useful for smoking reduction but not necessarily for complete cessation:

I’d go on the patches … and the inhaler … Then I’d cut down slowly as much as I could. (Female, 35-44, smoker) (Atkinson et al: 5 [51])

I’m going to buy one of they new electronic fags [ … ] Because a few of my friends have got them, and they do work, do you know what I mean. It’s like you can have a morning fag, and like a night time fag, but that helps you through the day if you’re out, [...] so I’m going to get one of them. (Female, 28, smoker) (Rowa-Dewar et al: 15 [50])

There were some differences between NRT and e-cigarettes when it came to potential harm reduction beliefs. Both Rooke et al. [49] and Thirlway [29] noted participants regarded NRT as more obviously a cessation aid than e-cigarettes, as vaping had connotations of recreation. This was reflected in Atkinson et al’s [51] study, which found that NRT was considered a cessation aid and medicinal product. The participants in Atkinson et al. [51] were generally negative about the potential for NRT to assist in temporary abstinence in the home. They felt that anything short of complete abstinence was not effective and using NRT while still smoking was ‘cheating’:

Well, I wouldn’t see much point in that [using NRT for temporary abstinence] to be honest if I was, if I was going to stop smoking, if I was going to use something like that I’d want to stop smoking completely, not just in the house. You know, because that way I wouldn’t be cheating going outside for a cigarette. (Female, 16-24, smoker) (p. 5)

One participant in Atkinson et al’s study did successfully use NRT for temporary abstinence in home, but the authors note that this was contrary to the prevailing experience of other participants. Despite these beliefs, many of the participants in Atkinson et al. [51] still indicated that they would try NRT sometime in the future to help them quit smoking. The participants in Rowa-Dewar et al’s [50] study of parents who smoke were more optimistic about the potential of e-cigarettes for temporary abstinence in the home:

Handy for you to cut down, because you can use that between … I smoke it in the house. (Female, 28, smoker) (p. 16)

Outside of these two studies, which directly looked at smoking and NCNP use in the home, using NCNP to cut down in homes was not evident in the other papers. Wiltshire et al. [53] did mention participants’ desires to cut down on smoking in the home but this was not related to NCNP use.

*(ii) NCNP alone perceived to have limited potential for smoking harm reduction.* A common theme among all the studies was that NCNP would not work unless people had motivation to quit smoking in the first place. Willpower was frequently mentioned as a more important resource for quitting than NCNP to the extent that some participants dismissed the value of NCNPs altogether:

While ‘patches’ might be used to initially stop smoking, like many interviewees, F35 felt that without ‘the willpower I don’t think they’re going to help you’. (Female, smoker) (Wiltshire et al: 299 [53])

I just don’t see the point. If you’re going to stop, use your willpower, don’t use some silly electronic device. (Female, 36, smoker) (Rowa-Dewar et al: 17 [50])

Building on the previous findings, these uncertain views demonstrated that alongside wider social, cultural and economic circumstances of participants, personal motivation was also a key factor in determining the perceived efficacy of NCNP.

## Discussion

Our review has sought to build on the wealth of research summarising the reasons for higher smoking prevalence among disadvantaged groups to synthesise the much smaller, emerging literature exploring perceptions and experiences of NCNP in relation to socioeconomic context. We identified two important gaps in this literature. First, none of our included studies focused on more socioeconomically advantaged groups which means that, for those concerned with inequalities, research is only providing a partial picture of one end of the spectrum. Second, there is a relative lack of sustained research exploring attitudes towards the different types of NCNP, with most papers identified in the initial stages of our review focussing on wider determinants of smoking prevalence but not NCNP. The relatively small number of included studies was somewhat surprising, due to the large field of qualitative literature on smoking and disadvantaged communities [28, 57] and the fact some forms of NCNP (NRT and smokeless tobacco) have been available for a long time.

Of the small number of papers that that focused specifically on the perceptions and experiences of socioeconomically disadvantaged communities on NCNP (i.e. those included in our study), we identified three major themes that shaped pessimistic and optimistic perceptions of NCNP: social, cultural and economic circumstance; relative advantage compared to smoking; and knowledge of relative harms (Table 4). The pessimistic accounts were dominant and contributed to an overall feeling that NCNPs were not widely considered for smoking harm reduction or cessation in socioeconomically disadvantaged groups. However, it is worth noting that, for the most part, the more optimistic findings came from studies focusing on e-cigarettes. Alongside pessimistic and optimistic perceptions, we also identified uncertain perceptions towards NCNP (Table 4), characterized by NCNP being considered as ineffective for harm reduction but not cessation; and NCNP being ineffective without individual motivation.

The relative dominance of pessimistic findings suggests that NCNPs are generally not seen as effective harm reduction or cessation products among socioeconomically disadvantaged groups. These pessimistic attitudes appeared largely to reflect the social and cultural circumstances of participants across the nine studies. This closely mirrors the literature on smoking among disadvantaged populations, which points to how aspects of people’s lives make it more difficult for them to avoid or quit smoking [30, 33, 34, 58]. The findings from this review go beyond linking smoking prevalence with stressful life circumstances, however, by highlighting that these circumstances are also not necessarily conducive to NCNP uptake. This supports recent research with smoking cessation practitioners in the UK who believe that limited income and social differences are major factors in lower uptake of e-cigarettes among disadvantaged people compared to more affluent smokers [59]. Nonetheless, some aspects of our findings suggest that e-cigarettes hold greater potential than NRT for some disadvantaged groups, including positive comments regarding use of e-cigarettes in the home in Rowa-Dewar et al’s [50] study and the potential for e-cigarettes to appeal to some young male smokers in Thirlway’s [29] study.

The more pessimistic perspectives on NCNP compared to smoking were linked to accounts of disliking NRT’s taste compared to smoking and through the embodied similarities of smoking and vaping which was perceived by some participants to maintain a ‘smoking’ habit or nicotine addiction. Conversely, there was also evidence that similarities could be viewed optimistically, as e-cigarettes could recreate existing smoking rituals and habits in ways NRT could not. Differences in smoking status may help explain this schism in opinion; recent ex-smokers in Rooke et al. [49] showed more trepidation concerning e-cigarettes than young male current smokers in Thirlway’s study [29], who noted the attractiveness and novelty of e-cigarette technology. Research has suggested that e-cigarettes may hold greater potential for harm reduction than NRT for reasons similar to those articulated by young men in Thirlway’s study: identity formation, socialising around vaping and vaping as a hobby [60, 61].

Pessimistic and optimistic beliefs regarding relative harm of NCNPs to smoking was another common theme across the studies. Potential product harms were a much more common concern for e-cigarettes, consistent with their novelty and relatively recent proliferation compared to the more established and medically licensed NRT. A simplistic interpretation of this finding might inadvertently reinforce a perception that disadvantaged smokers are somehow less able or willing to access health knowledge than others, informed by research which identifies lower health literacy in disadvantaged groups [62] Yet, Smith and Anderson [35] have suggested that disadvantaged groups are often well aware of the risks associated with unhealthy consumer products, such as cigarettes, and of the role that wider determinants (such as employment and housing) play in people’s decisions about health behaviours, such as smoking. All this suggests there is a need to improve the public’s knowledge about the risks of e-cigarettes compared to traditional, combustible tobacco products. Our findings suggest communication around the issue of continued nicotine addiction is a primary concern, a finding evident elsewhere irrespective of users’ SES [63, 64].

Although the uncertain opinions sat between more clearly pessimistic and optimistic views, they further reflect the contrast between the two views. They reflected more the nuance of how people form opinions and decisions about NCNP in relation to harm reduction and cessation. For example, the opinions that using NCNP in the home for temporary abstinence can be considered as ‘cheating’ reflects political and moral complexity of total abstinence versus harm reduction [65]. Likewise, the perceptions that willpower is essential for sustained uptake of NCNP reflects the possibility that factors other than socioeconomic circumstances are foundational for harm reduction or quitting. Arguably both these themes identified in the review are inextricably linked to SES, and so further research on how experiences of SES link in with attitudes to all types of combustible and NCNP is further required.

### Implications for current understandings and future research

Our review has two main interrelated implications for those working in policy and practice. First, it is important to consider how the social, cultural and economic circumstances of smokers may relate to their perceptions regarding products that are less harmful than combustible tobacco. This relationship is unlikely to be simple or necessarily fixed, meaning views cannot be assumed and must be explored. Second, an understanding of the differential significance of smoking and vaping among diverse social groups requires attention to the embodied and sensorial experiences of smoking and NCNP use. Building this understanding into policy and practice will potentially help improve the uptake and continued use of NCNP among smokers and so contribute to smoking harm reduction and cessation.

Importantly, our review points to an important avenues of future qualitative research that would enhance our understanding of how e-cigarettes and other NCNPs are being perceived and used among different social groups. Our review has suggested that the identity of those experiencing socioeconomic disadvantage is complex and intersects with other identities such as gender and parenthood. Therefore, future research has to finds ways to move away from monolithic constructs of identity (such as SES) and incorporate intersectional identities in order to understand the complicated and nuanced attitudes towards NCNP for smoking harm reduction or cessation. For example, gender dimensions were prevalent throughout Thirlway’s study [29] and seemed to influence participants’ attitudes towards e-cigarettes at least as much as SES. Although gender did not emerge as such an important component in the other papers, it is important to note that the study participants in some papers (e.g. Rowa-Dewar et al’s [50] and Atkinson et al’s [51]) were predominantly female parents and that these participants reported spending much of their time at home and undertaking care work, which further suggests that there are important gender dynamics to understanding experiences and perceptions of NCNP. This finding is relatively unsurprising given the gender dynamics and inequalities related to smoking [28]. Furthermore the similarity of embodied practice with smoking found throughout the e-cigarette studies, which influences attitudes towards, and hence uptake of, e-cigarettes needs to be further explored with an attention to habit, addition and social stigmas [20]. Nonetheless, for any researchers interested in understanding the likely (and potential) impact of NCNP on smoking related inequalities, further research on NCNP that takes account of SES is needed since this review demonstrates there is currently a lack of direct qualitative research on NCNP and SES.

### Strengths and limitations

A strength of this study was that the parent search strategy was methodologically inclusive and so enabled diverse articles to be included in our original search. Some systematic reviews of qualitative literature can limit search results due to the various definitions of qualitative methodological approaches. Through application of rigorous inclusion criteria we were able to identify a lack of qualitative literature directly addressing the equity impacts of NCNP.

One limitation with the study is that we excluded articles based on title and abstract screening, if they did not include direct reference to at least one form of NCNP. Since qualitative approaches to research on smoking regularly prompt participants to talk about harm reduction and cessation, it is likely that some excluded studies did contain some findings relating to NCNP and SES. However, it is unlikely that these would have been primary findings, so their exclusion is unlikely to weaken the insights provided by this review. Another limitation is that participants throughout the studies were possibly presenting a particular version of themselves as intending to quit smoking in order to achieve approval and avoid perceived judgement from interviewers. This was noted in Atkinson et al. [51], who point out that many of the participants in the study contradicted themselves over their quitting intentions.

It is also notable that the included studies did not make clear which types of e-cigarettes or NRT participants were using (and did not always discuss frequency of use). NCNP, especially e-cigarettes are increasingly diverse and future research will need to approach this diversity more carefully in order to reflect the different attitudes and practices of use of different product categories [66]. Additionally, the identification of studies that only focussed on socioeconomically disadvantaged groups meant that a comparison with other SES groups could not be undertaken. We suggest that this is an important avenue for future research because it is only really feasible to understand the likely equity impacts of NCNP if we have insights into the perceptions and experiences of groups across the SES spectrum.

A final, important point to note, is that further studies on this subject have been published since the initial search was carried out. In order to identify more recent trends in research we searched studies published since 2017 that cited our sample of nine. We found four papers that, based on title and abstract screening, would possibly be relevant to this review. Two of these studies reported research completed by one of the authors of this review, along with other colleagues [20, 67]. These studies are based on the same dataset as each other and start to answer some of our calls for further research, especially in terms of focussing more on the embodied and sensorial practices of NCNP use. The studies consider the social, cultural and ‘performative’ practices of vaping in relation to smoking among a sample of young people experiencing socioeconomic disadvantage in Scotland. The authors used a friendship group approach to data collection and found that, although e-cigarettes might have some potential to contribute to smoking harm reduction and cessation among this population, the experience of stigma associated with perceived addiction and the similarity of the embodied practices of smoking meant that there was limited enthusiasm for e-cigarettes. We also identified another study conducted by Thirlway [54]. Based on a new dataset, but utilising a similar ethnographic methodology, Thirlway builds on the findings of her previous study by further exploring the link between perceptions of addiction, morality and pleasure among a sample of working class smokers in Northern England. Thirlway’s more recent study represents a deepening exploration of the social and cultural experiences of socioeconomic disadvantage that can contribute to better understanding of how different NCNPs are perceived and taken up by different groups. The final identified study looked at the different perceptions of e-cigarettes among young people from contrasting high and low SES backgrounds in Liverpool [68]. An interesting finding that builds on our review, was that vaping was generally permitted indoors by parents, suggesting that e-cigarettes do have smoking harm reduction or cessation potential as Rowa-Dewar et al. [50] hinted. Of particular relevance to this review, this study found little difference between perceptions and practices of e-cigarette use between young people from higher and lower SES backgrounds, but the authors do note that limitations to their study including a very low number of regular e-cigarette users in their sample.

## Conclusion

This review highlights the importance of qualitative research in public health and tobacco control. While a recent commentary on e-cigarettes and public health has called for more objective approaches to considering the potential impact of devices [69], our lines-of-argument are crucial for reminding those working in the field that people do not always make health related decisions as ‘rational actors’ but are influenced by a wide array of social and cultural circumstances [70].

The dominance of pessimistic findings suggests that neither NRT nor e-cigarettes are currently perceived by those experiencing socioeconomic disadvantage to offer great potential for reducing smoking inequalities. Of particular significance was the largely similar attitudes to e-cigarettes and NRT evident across studies, which tempers the view that e-cigarettes currently provide a novel means of addressing smoking inequalities. Nonetheless, we did identify more optimistic perceptions with regards to e-cigarette use than NRT, such as the devices replacing similar habits and ritual, being used expeditiously and employing technologies that are attractive to some groups. All of this suggests there is some potential for e-cigarettes to achieve positive equity outcomes compared with NRT, provided interventions are able to take account of the importance of cost and of local modes of use in the context of SES and intersecting social dimensions, such as gender. The fact that our review identifies contrasting and uncertain perspectives about the relative harms of NCNP (especially e-cigarettes) is unsurprising given some of the research and media debates about these products, but it does suggest that there is a need to explore how to better communicate relative risks and harms in the context of scientific debate [71].

## Supplementary information

**Additional file 1.**

## Data Availability

A Supplementary File is available for this study: https://adobe.ly/3dmOlpx

## Abbreviations

NCNP: Non-combustible nicotine products
NRT: Nicotine Replacement Therapy
SES: Socioeconomic status
PRISMA-E: Preferred Reporting Items for Systematic Reviews and Meta-Analyses – Equity
CASP: Critical Appraisal Skills Programme
