# Supplementary material for: Inequalities, harm reduction and non-combustible nicotine products: a meta-ethnography of qualitative evidence
Source: BMC Public Health. 2020 Jun 15;20:943. doi: 10.1186/s12889-020-09083-9 (PMC7296947; doi:10.1186/s12889-020-09083-9)
Supplement: Supplementary file 1 — Additional file 1. [file 12889_2020_9083_MOESM1_ESM.docx]

# Inequalities, harm reduction and non-combustible nicotine products: a meta-ethnography of qualitative evidence

# Supplementary File

Mark Lucherini, Sarah Hill, Katherine Smith

## Contents: Pages

1. PRISMA-E Checklist 2-4

2. Example of search terms 5-6

3. Template of quality appraisal 7

4. Extended PRISMA diagram 8

5. Coding process for e-cigarette papers 9-18

6. Coding process for NRT papers 19-26

7. Synthesis table for 2^nd^ order codes, e-cigarettes and NRT 27-28

## PRISMA-E Checklist

*From:* Source: Welch V, Petticrew M, Tugwell P, Moher D, O'Neill J, Waters E, White H, and the PRISMA-Equity Bellagio Group. (2012) [PRISMA-Equity 2012 Extension: Reporting Guidelines for Systematic Reviews with a Focus on Health Equity](http://www.plosmedicine.org/article/info%3Adoi%2F10.1371%2Fjournal.pmed.1001333). PLoS Med 9(10): e1001333. doi:10.1371/journal.pmed.1001333

| **Checklist of Items for Reporting Equity-Focused Systematic Reviews** | | | |  |
| --- | --- | --- | --- | --- |
| **Section** | **Item** | **Standard PRISMA Item** | **Extension for Equity-Focused Reviews** | **Pg #** |
| **Title** |  |  |  |  |
| **Title** | 1 | Identify the report as a systematic review, meta-analysis, or both. | Identify equity as a focus of the review, if relevant, using the term equity | Title page, abstract, 3 |
| **Abstract** |  |  |  |  |
| **Structured summary** | 2 | Provide a structured summary including, as applicable: background; objectives; data sources; study eligibility criteria, participants, and interventions; study appraisal and synthesis methods; results; limitations; conclusions and implications of key findings; systematic review registration number. | State research question(s) related to health equity. | 2, 5-6 |
|  | 2A |  | Present results of health equity analyses (e.g. subgroup analyses or meta-regression). | 10-20 |
|  | 2B |  | Describe extent and limits of applicability to disadvantaged populations of interest. | 21-26 |
| **Introduction** |  |  |  |  |
| **Rationale** | 3 | Describe the rationale for the review in the context of what is already known. | Describe assumptions about mechanism(s) by which the intervention is assumed to have an impact on health equity. | 3, 26-28 |
|  | 3A |  | Provide the logic model/analytical framework, if done, to show the pathways through which the intervention is assumed to affect health equity and how it was developed. | 6-10 |
| **Objectives** | 4 | Provide an explicit statement of questions being addressed with reference to participants, interventions, comparisons, outcomes, and study design (PICOS). | Describe how disadvantage was defined if used as criterion in the review (e.g. for selecting studies, conducting analyses or judging applicability). | Abstract, 3, 6-7 |
|  | 4A |  | State the research questions being addressed with reference to health equity | 6 |
| **Methods** |  |  |  |  |
| **Protocol and registration** | 5 | Indicate if a review protocol exists, if and where it can be accessed (e.g., Web address), and, if available, provide registration information including registration number. |  | 6 |
| **Eligibility criteria** | 6 | Specify study characteristics (e.g., PICOS, length of follow-up) and report characteristics (e.g., years considered, language, publication status) used as criteria for eligibility, giving rationale. | Describe the rationale for including particular study designs related to equity research questions. | 6-7 |
|  | 6A |  | Describe the rationale for including the outcomes - e.g. how these are relevant to reducing inequity. | 8-9 |
| **Information sources** | 7 | Describe all information sources (e.g., databases with dates of coverage, contact with study authors to identify additional studies) in the search and date last searched. | Describe information sources (e.g. health, non-health, and grey literature sources) that were searched that are of specific relevance to address the equity questions of the review. | 7 |
| **Search** | 8 | Present full electronic search strategy for at least one database, including any limits used, such that it could be repeated. | Describe the broad search strategy and terms used to address equity questions of the review. | Supplementary material |
| **Study selection** | 9 | State the process for selecting studies (i.e., screening, eligibility, included in systematic review, and, if applicable, included in the meta-analysis). |  | 7-8 |
| **Data collection process** | 10 | Describe method of data extraction from reports (e.g., piloted forms, independently, in duplicate) and any processes for obtaining and confirming data from investigators. |  | 8-10 |
| **Data items** | 11 | List and define all variables for which data were sought (e.g., PICOS, funding sources) and any assumptions and simplifications made. | List and define data items related to equity,where such data were sought (e.g. using PROGRESS-Plus or other criteria, context). | Supplementary material |
| **Risk of bias in individual studies** | 12 | Describe methods used for assessing risk of bias of individual studies (including specification of whether this was done at the study or outcome level), and how this information is to be used in any data synthesis. |  | Supplementary material |
| **Summary measures** | 13 | State the principal summary measures (e.g., risk ratio, difference in means). |  | N/A |
| **Synthesis of results** | 14 | Describe the methods of handling data and combining results of studies, if done, including measures of consistency (e.g., I^2^) for each meta-analysis. | Describe methods of synthesizing findings on health inequities (e.g. presenting both relative and absolute differences between groups). | 8-10 |
| **Risk of bias across studies** | 15 | Specify any assessment of risk of bias that may affect the cumulative evidence (e.g., publication bias, selective reporting within studies). |  | 26-28 |
| **Additional analyses** | 16 | Describe methods of additional analyses (e.g., sensitivity or subgroup analyses, meta-regression), if done, indicating which were pre-specified. | Describe methods of additional synthesis approaches related to equity questions, if done, indicating which were pre-specified | N/A |
| **Results** |  |  |  |  |
| **Study selection** | 17 | Give numbers of studies screened, assessed for eligibility, and included in the review, with reasons for exclusions at each stage, ideally with a flow diagram. |  | 8, Supplementary file |
| **Study characteristics** | 18 | For each study, present characteristics for which data were extracted (e.g., study size, PICOS, follow-up period) and provide the citations. | Present the population characteristics that relate to the equity questions across the relevant PROGRESS-Plus or other factors of interest. | 8-10 |
| **Risk of bias within studies** | 19 | Present data on risk of bias of each study and, if available, any outcome level assessment (see item 12). |  | Supplementary material |
| **Results of individual studies** | 20 | For all outcomes considered (benefits or harms), present, for each study: (a) simple summary data for each intervention group (b) effect estimates and confidence intervals, ideally with a forest plot. |  | N/A |
| **Synthesis of results** | 21 | Present results of each meta-analysis done, including confidence intervals and measures of consistency. | Present the results of synthesizing findings on inequities (see 14). | N/A |
| **Risk of bias across studies** | 22 | Present results of any assessment of risk of bias across studies (see Item 15). |  | 26-28 |
| **Additional analysis** | 23 | Give results of additional analyses, if done (e.g., sensitivity or subgroup analyses, meta-regression [see Item 16]). | Give the results of additional synthesis approaches related to equity objectives, if done, (see 16). | N/A |
| **Discussion** |  |  |  |  |
| **Summary of evidence** | 24 | Summarize the main findings including the strength of evidence for each main outcome; consider their relevance to key groups (e.g., healthcare providers, users, and policy makers). |  | 21-29 |
| **Limitations** | 25 | Discuss limitations at study and outcome level (e.g., risk of bias), and at review-level (e.g., incomplete retrieval of identified research, reporting bias). |  | 26-28 |
| **Conclusions** | 26 | Provide a general interpretation of the results in the context of other evidence, and implications for future research. | Present extent and limits of applicability to disadvantaged populations of interest and describe the evidence and logic underlying those judgments. | 21-25 |
|  | 26A |  | Provide implications for research, practice or policy related to equity where relevant (e.g. types of research needed to address unanswered questions). | 25-26 |
| **Funding** |  |  |  |  |
| **Funding** | 27 | Describe sources of funding for the systematic review and other support (e.g., supply of data); role of funders for the systematic review. |  | 30 |

## Example of search terms

Search terms applied in MEDLINE

Smoking/tobacco/nicotine terms

smoking

tobacco

“tobacco use disorder”

nicotine

smoker OR smokers

cigar*

Non-combustible Nicotine Product Terms

smokeless

(smokeless or smoke-less or “smoke less”) AND tobacco

e-cig* OR “electronic cig*” OR electronic-cig*

ENDS OR “electronic nicotine delivery system*”

ANDS OR “alternative nicotine delivery system*”

snuff

snus

“nicotine replacement therap*” OR NRT

“nicotine replacement treatment*”

“nicotine replacement intervention*”

“heat not burn” OR “heat-not-burn”

vaporiser* OR vapouriser* OR vaporizer* OR vapourizer*

vape* OR vaping

(shop OR store) AND (vape* OR vapour OR vapor)

e-hookah* OR “electronic hookah*” OR electronic-hookah*

e-shisha* OR “electronic shisha*” OR electronic-shisha*

(nicotine OR tobacco) AND (patch* OR gum* OR inhaler* OR inhalator* OR lozenge* OR strip* OR stick* OR tablet* OR “nasal spray*” OR “mouth spray*” OR dissolvable OR oral OR dip* OR chew*)

Inequality terms

socioeconomic OR “socio economic” OR socio-economic

inequal*

disparit*

depriv*

disadvantage*

educat*

social AND (class* OR group* OR grade* OR context* OR status)

unemploy*

income

poverty

SES

demographic*

poor

equity

social AND (disadvant* OR exclusion OR excluded OR depriv*)

(Medline specific)

exp socioeconomic factors/

exp public assistance/

exp social welfare/

exp vulnerable populations/

1. **Template of quality appraisal**

The following template was used to assess the quality of all included papers. No papers were considered to be low quality (i.e. receiving a negative evaluation for any question. The only negative evaluation was for consideration of reflectivity, which was answered as ‘no’ for all but one paper (Thirlway 2017). However, this is not surprising given journal word restrictions and so was considered detrimental to the review. Thirlway’s study partially considered issues of reflexivity of completing the ethnographic observational methods. However, since other studies did not, issues of reflexivity have not been considered in the review.

| **Quality appraisal question** | **All papers** |
| --- | --- |
| Are research aims/objectives stated clearly? | Yes |
| Is the methodology appropriate for qualitative research and for the stated research aims? | Yes |
| Is the research design (ie methods) appropriate for the stated research aims? | Yes |
| Was the recruitment strategy appropriate? | Yes |
| Has the data collection process been adequately explained? | Yes |
| Have the methods of data analysis been explained in adequate detail? | Yes |
| Have the findings been presented with enough detail for clear conclusions about equity impacts by SES? | Yes |
| Have the authors considered issues of reflexivity in the research process? | No (partially for Thirlway) |
| Have ethical considerations been appropriately covered? | Yes |
| Have the limitations of the research been considered? | Yes |

1. **Extended PRISMA diagram**


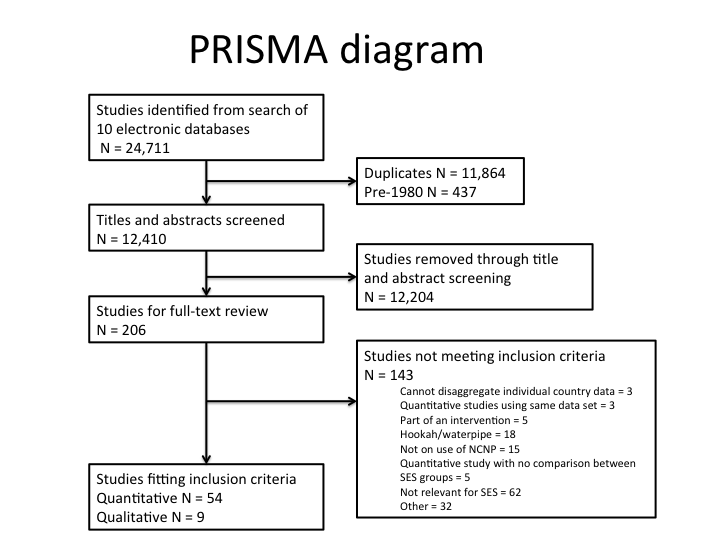


## Coding process for e-cigarette papers

### Table 1: Reasons why sustained e-cigarette use was not common among disadvantaged smokers

| **Example quote** | **1^st^ order concepts (initials of lead author of paper in which code occurs, followed by number of occurrences)** | **2^nd^ order concepts** | **Meta-codes** | **Lines of argument** |
| --- | --- | --- | --- | --- |
| “When I met Martin, unemployed and volunteering at a local community project, he was smoking thin ‘rollies’ as he worked. I asked if he had tried e-cigarettes, but they were ‘too expensive’, he told me. Although £10 would buy a starter tank and e liquid, smokers like Martin could get a week's worth of illicit rolling tobacco for the same money and could not risk such a large outlay on something that might not ‘work’ for him.” (Thirlway: 108) | Getting value for money (FT, 4)  Price of e-cigarette too high (FT, 2)  Awareness that e-cigarettes are not available on prescription (NRD, 1) | E-cigarettes expensive (NRD)  Thirlway also notes that the trial and error, hence expense, of finding a suitable e-cigarette was a barrier to uptake as people were restricted in “exploratory purchases” (p. 110) | Cost and price of e-cigarette not outweighing the relative advantage of using e-cigarette for harm reduction | Social, cultural and economic circumstances of low SES smokers not conducive to e-cigarette uptake or consistent use. |
| “Because […] there’s a wee bit too much stress in my life at the moment that I had to go back on the cigarettes” (Rooke et al: e63)  Kim (46), a cleaner who worked all hours to put her son through university, smoked thin cigarettes she rolled herself, except on nights out when she opted for ready-made cigarettes. Kim was a serial quitter and had tried everything to give up; she bought an e-cigarette in 2013 and this went well until ‘it rolled off the table and broke’ and she reverted to the pouch of illicit rolling tobacco tucked into the front pocket of her tabard. (Thirlway: 109)  “Giving up tobacco or taking up e-cigarettes can be more of a risk to moral identity than carrying on smoking" (FT: 111) | For women, family caretaking given greater priority that quitting imperatives (FT, 2)  E-cigarette use dominated by men and off-putting to women (FT, 1)  Stress leading to harm reduction plans being put off (NRD, 1; FT, 1)  Reverting to cigarettes when e-cigarette breaks down (FT, 1)  People with mental health problems depend on smoking (CR, 1)  Feeling resigned to continued smoking (CR, 1) | Gendered family roles make it more difficult for women to quit (FT)  Concern over public EC use (CR)  Everyday circumstances not conducive to cessation/conducive to smoking (FT, NRD | Social and cultural circumstances not conducive to quitting smoking |  |
| “But the e-cigs, like, you want to stop smoking, but you still want to … have something” (Rooke et al: e62)  “Them at Greendale [middle class area] haven't enjoyed themselves the way us lot have - I've got no regrets” (Thirlway p.110)  Finally, there were two distinct groups who did not interpret e-cigarettes as a useful technology: they fell at opposite ends of the smoker spectrum. The ﬁrst, while curious about e-cigarettes for their novelty value and interested in different packaging and ﬂavours, were happy smoking and did not see the point of switching to an e-cigarette. These smokers were relatively unconcerned about the health effects of smoking, not currently interested in quitting, and tended to be the youngest participants. There was also some confusion among these participants about whether e-cigarettes are ‘healthier’ than smoking” (Rooke et al: e63) | Continuing smoking part of working class identity (FT, 2)  E-cigarettes inconvenient to carry around (CR, 1)  Wanting to engage in harm reduction but not give up smoking entirely (CR, 2; FT, 2)  Not wanting to give up smoking (CR, 1)  Preferring the taste of a cigarette to an e-cigarette (CR, 1) | Smokers unconcerned with continued smoking (CR) | Not ready to give up smoking | E-cigarettes do not carry enough ‘relative advantage’ over smoking or other harm reduction products |
| “I don’t trust the electronic cigarettes, I just...I don’t think there’s been enough research on them. I think it’s making smokers more addicted to nicotine, [...]there’s loads of smoke that comes out, but that will be full of nicotine, so people are pufﬁng away at them, even if they’re smoking them in the car, it’s still nicotine, so you’re getting your child addicted to nicotine.” (Rowa-Dewar: 17)  “It’s not getting rid of the habit. [...] I’m still trying to persuade my husband to go on [nicotine replacement] patches, because I’m like, honestly, you’ve got to stop with that part of it [simulating smoking action]." (Rowa-Dewer: 18)  “I don’t feel like I’ve stopped smoking, I just feel like I smoke them instead” (Rooke et al: e63) | Similarity of vaping to smoking meaning that harm (both individual and environmental) is similar (CR, 1; NRD, 3; FT, 1)  Switching to an e-cigarette does not feel like quitting (CR, 3)  Similarity of hand-to-mouth action means e-cigarette will not help with harm reduction (CR, 1; NRD, 1) | E-cigarette similarities to smoking make people uneasy (including fear of continued nicotine addiction/smoking relapse) (FT, CR, NRD) | Embodied similarities between smoking and vaping as a negative |  |
| “What’s the point of smoking that? It’s not healthy for you. What’s the point? Those fags are just the same, they’re not healthy, you might as well just smoke fags.” (Rooke et al: e63)  “I think there’s just going to be another health scare with them soon enough because I think they’re disgusting, they really are, they’re just as bad as smoking.” (Rowa-Dewer et al: 17)  “I had one of those [e-cigarettes]… You didn’t know when to stop. At least with a cigarette you get to the end of it and you stub it out and that’s you for the next however long. But with that you could just sit and keep pufﬁng away.” (Rooke et al: e63)  “He’s on one of them, so I think she sees it as the [child] same motion but he doesn’t do it in front of her because he does it when he’s at work. He’ll go into the bedroom, same again as we used to do [with smoking], he won’t do it in front of her.” (Rowa-Dewer: 17) | Confusion about harm of nicotine (CR, 1)  Believing e-cigarettes to be bad for individual health (CR, 2; NRD, 2)  E-cigarettes seen as a potential future public health problem (NRD, 1)  Not knowing when to stop using and e-cigarette (CR, 2)  Adult/parental vaping as negative role-modelling for children (NRD, 2) | Unsure about e-cigarette safety (CR, NRD)  Unsure about EC safety/risks (CR)  EC similarities make people uneasy (including fear of continued/relapsed addiction) (NRD, CR, FT) | Beliefs about harm reduction | Lack of clear information about relative harms and regulation of e-cigarettes among disadvantaged communities |
| “Maybe if Boots [a UK pharmacy chain] did one or something you might trust it more, but all these…we’ve never even heard of this company. So I don’t know, it just seems a bit…” (Rooke et al: e64)  “I don’t trust the electronic cigarettes, I just...I don’t think there’s been enough research on them.” (Rowa-Dewer: 17)  “I’m now hearing there’s really bad stuff going...like they’re poisonous or this stuff that you’re inhaling.. .the liquid stuff is meant to be really, really bad for you. So, I need to stop” (Rowa-Dewar: 18) | Lacking trust in ‘informal’ e-cigarettes retailers (CR, 2)  Hearing ‘bad press’ about e-cigarettes (NRD, 2; FT, 1)  Scepticism due to lack of knowledge about e-cigarettes (CR, 1; NRD, 3) | Mistrust of ‘informal economy’ (CR) | Lack of trust in e-cigarettes manufacturers and retailers |  |

### Table 2: Understanding reasons why disadvantaged smokers would use e-cigarettes

| **Example Quote** | **1^st^ order concept** | **2^nd^ order concept** | | **Meta-concepts** | | | **Lines of argument** |
| --- | --- | --- | --- | --- | --- | --- | --- |
| When I last saw Adam, he was very proud of his latest, fourth-generation e-cigarette with wireless connectivity, and he told me that several of his friends had followed his example. (Thirlway: 108)  In one shop with a funky grafﬁti-style black and purple frontage and a ‘vape’ name, the owner Neil and a couple of customers, all men in their thirties with carefully-styled beards and intricate sleeve tattoos, were sampling a toffee donut ﬂavour and blowing out huge clouds of vapour. One of them told me he regularly travelled to the shop from another town twelve miles away, and had spent hundreds of pounds on different e-cigarette components and ﬂavours. (Thirlway: 109) | Pride in vaping gadgets (FT, 2)  Recreational vaping (FT, 2) | E-cigarettes used recreationaly to deny addiction (FT) | | Developing a ‘vaper’ identity** | | | The gadgetry and technology of vaping can be an attractive point for young men |
| “I use the eighteen milligram strength, tobacco ﬂavour, I can't see the point of the other ﬂavours I've tried ‘em, like had a puff off someone but it was horrible” (Thirlway: 109) | Disapproving of flavoured e-cigarettes (FT, 2) | E-cigarette use enhancing “local constructions of masculinity” through being a “badge of moral intent” among men with serious health problems (FT) | | | EC uses as a sign of masculinity in positive health decisions | | |
| “My husband, going back to it all, it’s the money. He sat down and thought about— when he was thinking about the vaporisers, he was like, it costs this much. But a lot of people he knew had stopped and they talked about the money.He said tome, it’s costing me £35 a week in tobacco, and I was in denial. We can’t afford to smoke because I’m not working and we’ve got the girls but we were like, our savings are getting less and less. [.. .]The vaporisers have been good in that way”. (Rowa-Dewar: 17) | E-cigarettes saving money (NRD, 1; CR, 1) | | | | | | E-cigarettes as an alternative to smoking can have a positive financial impact |
| Using the informal economy to "demonstrate moral worth in relation to the moral problems of addiction and expenditure on the self" (FT: 111) | | | Utilisation of ‘informal e-cigarette economy’ to save money and ‘recover agency’ and ‘moral worth’ among working class community | | | | Resisting and creating an alternative functional use of e-cigarettes to ‘lifestyle’ and ‘hobbyist’ e-cigarette culture in working class communities*** |
| “They’re more satisfying. Much more satisfying. I think because, see when you take a puff, it actually feels like, you used to get that kind of hit off a cigarette when you took a puff off the cigarette, you get that sensation from the e-cig” (Rooke et al: e62) | The ‘hit’ from vaping as a satisfying alternative to smoking inhalation (CR, 1) | | Similarities to smoking good (CR, NRD) | Embodied similarities between vaping and smoking as a positive | | | E-cigarettes have some ‘relative advantage’ over cigarettes. |
| “You’re still smoking nicotine, but you’re not smoking tar and you’re not making your lungs… you’re not making your lungs get covered in tar.” (Rooke et al: e63) | Belief that e-cigarettes are not as harmful as cigarettes (CR, 3) | | E-cigarettes believed to be less harmful than CTS (NRD)  Some current smokers unconcerned about continued nicotine dependence (CR) | | | Accepted knowledge about relative harm reaching most disadvantaged | |

### Table 3: Perceptions and approaches to harm reduction strategies among disadvantaged smokers

| **Example quote** | **1^st^ order coding** | **2^nd^ order coding** | **Meta-themes** | **Lines of argument** |
| --- | --- | --- | --- | --- |
| “Because I have been thinking about buying one after Christmas because that’s a good way to help you stop” (Rowa-Dewar et al: 15) | Thinking about or using an e-cigarette to stop smoking (NRD, 3; FT, 1)  Using e-cigarettes to eventually quit nicotine addiction (NRD, 1; FT, 1) | E-cigarettes having potential for HR outcomes (CR, NRD) | E-cigarettes as a serious and effective cessation aid | E-cigarettes useful for smoking reduction but not necessarily for complete smoking cessation |
| “As part of our cutting down, bought the electronic cigarettes” (Rooke et al: e64)  “I’m going to buy one of they new electronic fags.[.. .]Because a few of my friends have got them, and they do work, do you know what I mean. It’s like you can have a morning fag, and like a night time fag, but that helps you through the day if you’re out, [...]so I’m going to get one of them” (Rowa-Dewar: 14)  He was using an e-cigarette when I ﬁrst met him in 2013, but a plastic box containing ﬁlter tips, tobacco pouch and cigarette papers sat on the counter at the project where he volunteered, and he was often out on the pavement smoking. In November 2014, when I saw Mark helping to unload a van despite his bad back, he assured me that the recessed ﬁlter of the (illicit) cigarettes he was smoking made them safer. In any case, he told me, he was going to the market that week to buy a new e-cigarette (Thirlway: 108) | Thinking about or using an e-cigarette to cut down but not necessarily with the intention of stopping (CR, 1; NRD, 3; FT, 2)  Dual using e-cigarettes and combustible tobacco (NRD, 1; FT, 2) | Unsure about e-cigarettes’ role in HR outcomes (CR) | |  |
| "handy for you to cut down, because you can use that between, when you’re [...]I smoke it in the house." (Rowa-Dewar: 16)  “[I smoke normal cigarettes] when I’m out. Like I’ve got fags on me just now, because I’m out, and I’m not with [my son].But usually when I’m with him and I’m in the house, it’s usually my electric fag that I use. But it doesn’t help that he’s got a bad chest as well, so, like, the electric fag won’t harm him, so I thought, well, if I use that in the house, it’s not got any of the fumes or anything in it; it won’t harm his chest. So I started using that.: (16) | Protecting others (especially children) from ETS with e-cigarettes (NRD, 3)  Declaring intention for smokefree home (NRD, 2)  Thinking about or using an e-cigarettes expeditiously (NRD, 1) | EC having potential for HR (CR, NRD) | Smokers going in and out of e-cigarette use |  |
| “…thinking rationally about it right now, it would just be a slippery slope right, right down to fags” (Rooke et al: e62) | EC similarities make people uneasy (including fear of continued/relapsed addiction) (FT, NRD, CR) | E-cigarettes seen as a possible gateway to smoking relapse | E-cigarettes useful for reducing smoking in the home (temporary abstinence) | E-cigarettes useful for smoking reduction but not necessarily for complete smoking cessation |
| “I just don’t see the point. If you’re going to stop, use your willpower, don’t use some silly electronic device and make somebody else a ton of money, you know.” (Rowa-Dewar: 17)  When I saw Craig's mother a year later, she told me proudly that he had stopped smoking in order to pass soldier selection ﬁtness tests and join the British Army. I was not surprised to hear, given his views about e-cigarettes, that Craig had used pharmacotherapy (NRT or varenicline) to quit. (Thirlway: 108) | Perceiving other forms of harm reduction product as more effective (NRD, 1; FT, 1)  Perceiving willpower as more crucial for quitting than e-cigarettes (NRD, 1; FT, 1) | Unsure about EC role in HR outcomes (CR)  EC similarities make people uneasy (including fear of continued/relapsed addiction (FT, CR, NRD) | | E-cigarettes alone have limited potential for smoking harm reduction |

## Coding process for NRT papers

### Table 1: Reasons why sustained NRT use was not common among disadvantaged smokers

| **Example quote** | **1^st^ order concepts (initial indicate paper concepts appear in + number of occurrences)** | **2^nd^ order concepts** | **Meta-concepts** | **Lines of argument** |
| --- | --- | --- | --- | --- |
| ‘If someone gave me $50 a week to quit, yeah’ (Bonevski et al: 414)  “I’d take it for sure.... If you said patches they are for free or $2.50, I’m telling you there would be way more people having a crack at giving up” (Bryant et al 2011: 5)  ‘I could try the patches again but they’re too expensive’ (Wiltshire et al: 299)  I would rather spend £15 on cigarettes, in case I had the patches and needed cigarettes as well. (Wiltshire et al: 299)  It is also possible that the failure to engage with the concept of temporary abstinence in our study group could be partly explained by the relative lack of exposure to situations where the smokers have to abstain from smoking. Many of our participants were unemployed or homemakers, and it is likely that they spend less time in situations of enforced temporary abstinence from smoking than more advantaged households with higher employment rates where smokers are potentially more used to the concept of enforced temporary abstinence and could then transfer this approach to their homes (Atkinson et al: 6)  ‘[We’re] not comfortable living here... I’m unemployed... Stress levels have been very high [and] we have noticed we smoke a lot more’ (Wiltshire et al: 299) | Intention to quit for financial incentive (BB, 3)  Open to trying NRT if it were free or cheaper (BB, 2, JB2011, 2)  Wariness of spending money on NRT that might not work (SW, 2; JB2011, 2)  Price of NRT too high (SW, 2; JB2011, 1, BB, 2; ER, 4) | Everyday circumstances not conducive to quitting/conducive to continued smoking (OA, SW)  Quitting smoking just as expensive as continuing smoking (BB)  Readiness to use NRT among smokers if less expensive (BB) | Cost of NRT putting people off | Social, cultural and economic circumstances of low SES smokers not conducive to NRT uptake |
| [the] packet of fags was there and I just picked it up and lit a fag and that was it’ (Wiltshire et al: 297)  The area you live in and all that kind of thing affects you as well... They’re knocking all the houses down round about here and we’re going to be getting a nice, brand new built house with a front and back door, and I think that might have an inﬂuence on both of us giving up (Wiltshire et al: 299)  [I] was off the cigarettes for about seven and a half months... on patches. I went through a bad period and started back up again, and I stopped smoking again... Once again with the chewing gum... Went through another bad patch, started back up again and I’ve not long since started back up, after going through another bad patch. (Wiltshire et al: 298)  ‘the job at the time made me start again’ (Wiltshire et al: 296)  ‘cigarettes sort of calm you down’ (Wiltshire et al: 297)  “I gave it away and then 7th of July last year, went off for four months and then me nerves played up on me so I went back on” (Bryant et al 2011: 4)  'You finish washing up and you have a fag, when you have a cup of tea you have a fag, when you sit down and watch Neighbours you have a fag ...' (Roddy et al: 3)  '...I know when I stop smoking the weight goes on.' (Roddy et al: 4) | Cigarettes easily available through friends and socialising (SW, 1; ER, 3)  Feeling that impoverished area makes quitting more difficult than a more affluent area would (SW, 1)  For women, family caretaking given greater priority than quitting imperatives (SW, 1)  Going on and off NRT due to stress (SW, 2)  Lapse in quit attempt due to stress (not explicitly using NRT) (SW, 6)  NRT not satisfying stress like a cigarettes does (SW, 2)  Smoking as a natural part of culture and social life (SW, 5; JB2011, 5; ER, 5)  Smoking helps relieve stress (SW, 8; JB2011, 3; ER, 2)  Never being the right time to quit (JB2011, 1)  Fear of gaining weight when quitting (JB2011, 2; ER, 1) | Social and cultural circumstances not conducive to quitting (OA, SW, BB/JB, ER) | |  |
| “It’s like alcoholics, I, I attribute it to that – alcoholics, if you say to an alcoholic, cos I have an alcoholic in my family, me father. If you say to the alcoholic, we’re not saying you can’t drink, we’re just saying you can drink there – he’ll drink everywhere and I think that would be the same with smoking” (Atkinson et al: 4)  I was moody, I was moaning, I was arrogant. You get these mood swings and temper and everything, because you’re craving for a cigarette. The slightest thing that she would do wrong I would shout at her. (Wiltshire et al: 298) | CTS too great an addiction to easily quit (SA, 2; SW, 3; JB2011, 4; ER, 7)  Doubtful that smoke-free house is feasible given smoking addiction and behaviour (SA, 3)  Not being happy with personality and behaviour when abstinent (SW, 2; ER, 1) | Feeling that smoking is too great an addiction to quit easily (SW, BB/JB)  Feeling unsupported to quit (BB/JB) | Smoking an ingrained and normal part of life |  |
| Similarly, F21 described how her friend had tried to quit using patches: ...but she’s started smoking again... It’s a bad situation, she’s had a lot happening to her and I feel to try and stop smoking at this time was stupid. (Wiltshire et al: 298)  “I think a lot of it is about you wanting to at that time and the will power and you can sit and you can say, oh I've had these patches on for 2 weeks and not done anything but if you're wearing the patches and still constantly doing your same habit with your cigarettes, I mean, you're not going to be able to give up. I think it's got a lot to do like, if I want to give up and I take everything, you know, and I try every single thing but then at the same time I still keep my cigarettes in my everyday routine I'm not gunna give up am ?” (Atkinson et al: 5)  'I know all the statistics, I know all the stuff that's there to know and I'm a strong willed person, anything else I've overcome in my life, massive problems and all the rest of it, but one of the things has been a constant through my life is the smoking, it's that element of .... having tried and failed.' (Roddy et al: 4) | Lacking strong motivations to quit (SW, 1; JB2011, 1)  Never being ‘the right time to quit’ (SW, 1) | Unconcerned about continued smoking (SW, BB/JB) | Feeling that continued smoking is inevitable |  |
| ‘Like seriously if you were given money they’d more than likely use to go and buy cigarettes and tell you they are not smoking and get a mouth freshener’. (Bonevskie et al: 414)  “I had the gum years ago.. .but erm and it tastes horrible, it tastes like erm, I know when people say it's not like that now but it used to taste like you were chewing an ashtray” (Atkinson et al: 4)  “I tried it once, none of 'em worked so there's no point” (Atkinsone et al: 5)  ‘bad smokers cough’ was relieved by smoking it ‘doesnae [does not] give me the incentive to stop’. (Wiltshire et al: 296)  ‘I know it’s bad for me and everything like that, but I do enjoy it’ (Wiltshire et al: 297)  “I don’t know how much of sort of my anxiety with it is about, I s'pose m’ obviously it's a lot to do with the nicotine but also the sort of give myself five minutes space or fresh air or out on your own sort of thing so I don’t know how, cos I've never really tried them I don’t know how much my sort of anxiety’s to do with just wanting to sort of get out and have, do that ritual or if it's actually just the nicotine sort of thing yeah” (Atkinson et al: 5)  'You're going to die whether you smoke or not.' (Roddy et al: 4) | Seeing others struggle with NRT (SW, 1)  Not wanting to quit (BB, 1)  NRT having an unpleasant taste (SA, 1)  NRT not working (SA, 2)  Stopping smoking seemingly bad for health (SW, 1)  Smoking for pleasure (SW, 2; ER, 1)  NRT not helping with ritual aspect of smoking (SA, 2; SW, 2)  Unconcerned about continued smoking (ER, 1) | Health imperatives not a good enough reason to stop (SW)  (sub-code of unconcerned about continued smoking) | NRT do not carry enough ‘relative advantage’ over smoking | |
| “I kind of understand it.. .but then on the other side of it I think because it's nicotine replacement so how is it gunna help you stop if it's still giving you the nicotine” (Atkinson et al: 4)  “I don’t know, I just can’t see it [use of NRT] cos it might, you don’t know why, people don’t know the effects, it’s going to make you feel odd: (Atkinson et al: 5)  In our study, few caregivers who smoked in the home appeared to link our discourse about temporary abstinence to their children’s health. Instead, the emphasis amongst our participants seemed to be around personal goals, such as cutting down or the desire to stop smoking completely, rather than temporarily abstaining from smoking in their homes to benefit their children (Atkinson et al: 6) | Confusion about nicotine harm (SA, 2)  Lack of knowledge about NRT relative harms (SA, 2)  Not receiving enough support to quit (JB2010, 7; JB2011, 1; ER, 7) | Negative effects of ETS underestimated (OA) | Beliefs about harm reduction | Lack of clear information about relative harm of NRT among disadvantaged communities |

### Table 2: Perceptions and approaches to harm reduction strategies among disadvantaged smokers

| **Example quotes** | **1^st^ order concepts** | **2^nd^ order concepts** | **Meta-concepts** | **Lines of argument** |
| --- | --- | --- | --- | --- |
| “Well, I wouldn’t see much point in that [using NRT for TA] to be honest if I was, if I was going to stop smoking, if I was going to use something like that [NRT] I’d want to stop smoking completely, not just in the house. You know, because that way I wouldn’tbe cheating going outside for a cigarette or – I really do need to stop smoking myself, for myself, for the children cos that way I’m going to be here longer hopefully” (Atkinson et al: 5)  I’ll say oh I want a fag anyway. So I just think it's a waste, in my mind I think it's a waste of time” (Atkinson et al: 5)  “I kind of understand it.. .but then on the other side of it I think because it's nicotine replacement so how is it gunna help you stop if it's still giving you the nicotine” (Atkinson et al: 4) | Anything short of complete abstinence not thought to be effective (SA, 3)  Feeling NRT is a ‘waste of time’ (SA, 2, JB2011, 1)  Doubtful about NRT for harm reduction (SA, 5, JB2011, 1, ER, 2)  ‘cheating’ when using NRT but not quitting (SA, 1) | NRT not useful (SA, SW) | NRT not seen as genuinely effective cessation aid |  |
| “I’d love to start using like stuff like that to quit altogether as well” (Atkinson et al: 5)  “Possibly to do it in like a two-step phase, to do that one first and then after a while stop altogether.. .Make the smokefree house permanent, smoking outside but then, erm, after a while just give up totally” (Atkinson et al: 4) | Thinking about or using NRT to stop smoking (OA, 3, JB2011, 1)  Thinking about or using NRT to cut down to quit (OA, 3) | NRT considered useful HR outcomes(OA)  Quitting intentions of participants unclear (SW) | Smokers going in and out of NRT use | NRT seen as useful for smoking reduction and temporary abstinence but attempts are not lasting and participants are doubtful about potential for complete cessation |
| “I'd erm go on the patches ... and the inhaler. So I've got something in my hand .. . Then I'd cut down slowly as much as I could” (Atkinson et al: 5) | Thinking about or using NRT to cut down but not necessarily to quit (SA, 1) | NRT not useful for HR outcomes (OA, SW, BB) |  |  |
| Just thought it would be easier instead of smoking inside, it’s easier just to go outside and then your son’s safe and your kids are safe then if you don’t smoke in front of them … That’s when [I had cravings] I used to chew the, er, chewing gum as well. A couple of hours it used to work then I’d just think, used to think go outside and have one” (Atkinson et al: 5 | Declaring intention for a smoke-free house (SA, 1)  Successfully using NRT for temporary abstinence in the home (OA, 1) | NRT can cut down on ETS (SW) | NRT useful for temporary reduction in the home |  |
| While ‘patches’ might be used to initially stop smoking, like many interviewees, F35 felt that without ‘the willpower I don’t think they’re going to help you’ (Wiltshire et al: 299)  “I just don’t think it works. I think it's more willpower.. . And if you, if you’ve got the willpower you’ll say no, I'm no, I'm not going to. See I haven’t and I’ll say oh I want a fag anyway. (Atkinson et al: 5) | Perceiving willpower as important for cessation rather than NRT (SA, 4; SW, 4) | NRT not useful for HR outcomes (SW) | NRT alone have limited potential for smoking harm reduction | |

## Second order codes synthesis tables

|  | Everyday circumstances not conducive to cessation/conducive to continued smoking | Smokers unconcerned with continued smoking | E-cigarettes having potential for any harm reduction outcome | Unsure about e-cigarette role in harm reduction outcomes | E-cigarettes believed to be less harmful than combustible tobacco smoking | Unsure about e-cigarettes safety | E-cigarettes are expensive | People mistrustful of e-cgiarette informal economy | Utilisation of informal e-cigarette economy to save money and ‘recover agency’ and ‘moral worth’ among working class community | E-cigarette similarity to smoking helpful for cessation | EC similarities make people uneasy (including fear of continued/relapsed addiction) | Concerned about public e-cigarette use | | E-cigarette use a sign of maturity in positive health decisions | E-cigarette difficult to get started with | Gendered family roles make it more difficult for women to quit | E-cigarette used recreationally |
| --- | --- | --- | --- | --- | --- | --- | --- | --- | --- | --- | --- | --- | --- | --- | --- | --- | --- |
| Thirlway | X |  |  |  |  |  |  |  | X |  | X |  | X | | X | X | X |
| Rooke et al | X | X | X | X |  | X |  | X |  | X | X | X |  | |  |  |  |
| Rowa-Dewar et al | X |  | X |  | X | X | X |  |  | X | X |  |  | |  |  |  |

### Table 1: E-cigarettes

### Table 2: NRT

|  | NRT as useful for harm reduction outcomes | NRT not useful for harm reduction outcomes | Negative effects of environmental tobacco smoke possibly underestimated | NRT possibly useful for temporary abstinence in home | Everyday circumstances not conducive to quitting/conducive to continued smoking | Quitting intentions of participants unclear | Quitting smoking just as expensive as continuing smoking | Readiness to use NRT among smokers if less expensive | Unconcerned about continued smoking | Financial incentives might not be effective to encourage cessation | Feeling smoking is too difficult to quit | Feeling unsupported to quit |
| --- | --- | --- | --- | --- | --- | --- | --- | --- | --- | --- | --- | --- |
| Atkinson et al | X | X | X |  | X |  |  |  |  |  |  |  |
| Bonevski et al  Bryant et al 2010, 2011 |  | X |  |  | X |  | X | X | X | X | X | X |
| Wiltshire et al |  | X |  | X | X | X |  | X | X |  | X |  |
| Roddy et al |  | X |  |  | X |  |  |  |  |  |  | X |
